# The transnational Sowa Rigpa industry in Asia: New perspectives on an emerging economy

**DOI:** 10.1016/j.socscimed.2019.112617

**Published:** 2020-01

**Authors:** Stephan Kloos, Harilal Madhavan, Tawni Tidwell, Calum Blaikie, Mingji Cuomu

**Affiliations:** a Institute for Social Anthropology, Austrian Academy of Sciences, Austria; b Indian Institute of Science Education and Research, Thiruvananthapuram, India; c Center for Healthy Minds, University of Wisconsin-Madison, United States; d Tibet University of Tibetan Medicine, Lhasa, China

**Keywords:** Sowa Rigpa, Tibetan medicine, Pharmaceutical industry, Government policy, China, India, Mongolia, Bhutan

## Abstract

This article advances the hypothesis that “traditional” Asian pharmaceutical industries are rapidly growing in size and prominence in contemporary Asia, and identifies a lack of empirical data on the phenomenon. Addressing this gap, the article provides a quantitative outline and analysis of the Sowa Rigpa (Tibetan, Mongolian and Himalayan medicine) pharmaceutical industry in China, India, Mongolia and Bhutan. Using original data gathered through multi-sited ethnographic and textual research between 2014 and 2019, involving 232 industry representatives, policy makers, researchers, pharmacists and physicians, it assembles a bigger picture on this industry's structure, size and dynamics.

Revealing a tenfold growth of the Sowa Rigpa pharmaceutical industry in Asia between 2000 and 2017, the study supports its initial hypothesis. In 2017, the industry had a total sales value of 677.5 million USD, and constituted an important economic and public health resource in Tibetan, Mongolian and Himalayan regions of Asia. China generates almost 98 percent of the total sales value, which is explained by significant state intervention on the one hand, and historical and sociocultural reasons on the other. India has the second largest Sowa Rigpa pharmaceutical industry with an annual sales value of about 11 million USD, while sales values in Mongolia and Bhutan are very low, despite Sowa Rigpa's domestic importance for the two nations.

The article concludes with a number of broader observations emerging from the presented data, arguing that the Sowa Rigpa pharmaceutical industry has become big enough to exert complex transformative effects on Tibetan, Mongolian and Himalayan medicine more generally. The quantitative and qualitative data presented here provide crucial foundations for further scholarly, regulatory, and professional engagement with contemporary Sowa Rigpa.

## Highlights

• Provides the first quantitative overview of Asia's Sowa Rigpa pharmaceutical industry.
• Assesses the size, structure and dynamics of this industry in China, India, Mongolia and Bhutan.
• Shows how the industry has grown by a factor of 10 since 2000, becoming a major economic and health resource.
• Argues that government policies have significantly shaped development trajectories.
• Provides baseline data of value to researchers, industry actors and policy-makers.

Sowa Rigpa (Tib. *gso ba rig pa*) literally means “the science of healing” and counts as one of Asia's four “great scholarly medical systems” next to Chinese medicine (TCM), Ayurveda, and Unani. Best known as “Tibetan medicine”, it actually constitutes a family of regional medical traditions – including Mongolian, Bhutanese, and Himalayan Amchi medicine – that are all based on a singular standard treatise, the *Gyüzhi* or *Four Tantras* (Craig and Gerke, 2016). Sowa Rigpa emerged in Central Tibet between the seventh and twelfth century CE from Tibetan, Indian, Chinese, Persian, and Central Asian medical knowledge (Kilty, 2010; Yang, 2014). Between the thirteenth and seventeenth century CE it spread, together with Tibetan Buddhism, throughout the Tibetan plateau (Wangdü, 2016), Mongolia (Bold, 2009) and the Himalayan range (e.g. Wangchuk, 2008; Kloos and Pordié forthcoming). Relying on a complex formulary of multi-ingredient drugs (using herbs, minerals, metals, and animal products), Sowa Rigpa served as the sole pharmaceutical health resource for an area roughly the size of Europe until the early/mid-twentieth century, when biomedicine was introduced. Decades of official suppression in Tibet, Mongolia, and Siberia (e.g. Hofer, 2018; Kloos forthcoming), and governmental neglect in Himalayan areas (e.g. Craig, 2012; Pordié and Kloos forthcoming) ensued. In the 1980s and 90s, Sowa Rigpa began to reemerge as an increasingly popular primary health resource (e.g. Janes, 2002; Craig and Adams, 2008; Hofer, 2018; Blaikie, 2019) and an important placeholder for various national and ethnic identities (Janes, 1995; Janes and Hilliard 2008; Kloos, 2017a). Sowa Rigpa's growth and development since then was shaped by processes of modernization, globalization, and commercialization in all its locations (e.g. Adams, 2001; 2007; Janes, 1999, 2001, 2002; Janes and Hilliard 2008; Saxer, 2013), culminating in the emergence of a Sowa Rigpa industry from the late 1990s and early 2000s.

This recent and ongoing industrialization arguably constitutes the biggest transformation that Sowa Rigpa has undergone in the past several hundred years. Never before have Sowa Rigpa medicines been produced in such quantities and consumed by so many patients, in as many parts of the world, as today. Sowa Rigpa's dramatic transition from a marginal and regionally diverse family of medical traditions, each concerned with its own survival, to a transnational pharmaceutical industry struggling to meet growing demands, has coincided with a global trend of pharmaceuticalization (Kloos, 2019). Apprehending Tibetan, Mongolian, Bhutanese, or Amchi medicine today, then, requires an accurate understanding of the Sowa Rigpa pharmaceutical industry. Contrary to most existing scholarship, this means considering economic aspects as central rather than peripheral matters. While Sowa Rigpa remains a rich and diverse field of medical knowledge and practice that extends far beyond economics, questions of growth, value and scale simply cannot be ignored any longer. It makes a sociocultural, political, and public health difference whether a medicine is practiced only in remote rural communities or across large parts of Asia and the world, and whether it is merely surviving or growing into a multi-million-dollar industry. Ideally, this kind of data should provide the basic foundations for any scholarly, regulatory, or professional engagement with contemporary Asian medicines, including Sowa Rigpa. Yet despite a growing body of clinical, pharmacological, and social science research on related topics, such data have not been available so far. In order to address this gap, this article presents a quantitative outline of the Sowa Rigpa pharmaceutical industry in Asia and analyses its structure, size and dynamics. It thereby offers a new perspective for Tibetan medicine studies and enables meaningful comparison with other “traditional” pharmaceutical industries in Asia and beyond.

Various transformations related to Sowa Rigpa's industrialization have been relatively well studied, including its official status and historiography (Blaikie, 2016; Kloos, 2016), nomenclature (Craig and Gerke, 2016), training modalities (Craig, 2012; Pordié and Blaikie, 2014; Tidwell, 2017), clinical and pharmaceutical practices (e.g. Gerke, 2011; Schrempf, 2015; Tidwell and Nettles, 2019), and medical knowledge (Prost and Hermans, 2006; Adams and Li, 2008). However, the pharmaceutical industry in and of itself has only recently begun to attract serious scholarly attention (Saxer, 2013; Kloos, 2016; 2017b, 2019; Madhavan, 2017), which underscores the need for a firmer quantitative basis on which subsequent research can build. From these initial studies, we know that state economic and health policies play key roles in Sowa Rigpa's industrial development in almost all locations, beginning with its legal recognition. Today, Sowa Rigpa is officially integrated into the national health care systems of China, India, Mongolia, and Bhutan, which are also the main sites of its industry. In each location, Sowa Rigpa has become a significant domain of economic activity, driven by mass production of pharmaceuticals and health products from natural ingredients identified in Sowa Rigpa texts. Thus defined, the Sowa Rigpa industry can be seen as a transnational pharmaceutical assemblage (Kloos, 2017b), held together not only by a common theoretical and literary core, a shared diagnostic and therapeutic repertoire, and to some extent a common *materia medica*, but also by a large network of formal and informal flows of people, substances, knowledge, values, and discourses. While its heterogeneous elements (old and new) do not necessarily all fit together smoothly, they nevertheless constitute a larger whole with its own characteristics and dynamics. By focusing on the size, structure, and dynamics of the Sowa Rigpa industry, a bigger picture of this pharmaceutical assemblage emerges.

## 1 Methodology and limitations

This quantitative overview is based on empirical and textual research conducted by the authors in China, India, Mongolia, and Bhutan (and to a lesser extent Nepal, Russia, and Poland) between 2014 and 2019. Given the emerging and partially unregulated nature of the industry, and the generally difficult access to company accounts, in most cases the only feasible way to obtain reliable data was through ethnographic methods, that is, qualitative interviews and participant observation based on long-term research experience, local linguistic and cultural competence, and strong professional networks. This approach provided unique access to internal written sources where they existed, and generated detailed quantitative material on pharmaceutical production where they did not. It also yielded the sociocultural, political, and historical data necessary to properly interpret economic figures and situate them in a broader context. Mixed qualitative and quantitative data – interview transcripts, observation notes, official documents, internal data sheets, published material – obtained from different sources were triangulated to ensure validity, compiled and aggregated into larger analytic units, and finally checked by Sowa Rigpa experts and stakeholders to ascertain whether the results matched their own local knowledge and general estimations. During the study timeframe, the authors conducted a total of 338 interviews with 232 key actors (industry representatives, policy makers, researchers, pharmacists, physicians) at 140 Sowa Rigpa institutions and pharmaceutical producers in the four countries, and collected, translated and analyzed 62 unpublished documents in addition to cited scholarly publications and media reports. Not included in these numbers are 35 interviews with 24 key actors at 14 Sowa Rigpa institutions and pharmaceutical producers in Russia, Nepal, Poland, Switzerland and Austria, which yielded valuable additional data.

**Table:** 

Country	Interviews	Interviewees	Institutions	Documents
China	183	141	89	7
India	104	56	32	40
Mongolia	41	30	17	12
Bhutan	10	5	2	3
**TOTAL**	**338**	**232**	**140**	**62**

Wherever possible, we assembled economic data directly from internal accounts, balance sheets, or annual reports of larger Sowa Rigpa companies and institutions: in China, from eight of the most important Tibetan medicine producers (Cheezheng, Arura, Ganlu, Shongpalhachu, Jiumei, Shigatse Zangnuo, Nyalam Tibet Phenhou, and the Qinghai Provincial Tibetan Medical Hospital); in India from the Dharamsala Men-Tsee-Khang, Chagpori Tibetan Medical Institute, and Ladakh Amchi Sabha; and in Bhutan from Menjong Sorig Pharmaceuticals. We obtained original government policy documents regarding the regulation and development of the Sowa Rigpa industry on national and provincial levels in China and Mongolia, as well as unpublished aggregated government figures on industry values for Mongolia, the Tibetan Autonomous Region (TAR), the Inner Mongolia Autonomous Region (IMAR), and the cities of Tongliao (IMAR) and Fuxin (Liaoning province). Scholarly publications, mostly in Chinese language, constituted a third important source of industry values and overviews of policy development for Sowa Rigpa in China in general (e.g. Zhugen et al., 2015), Tibetan medicine in China (e.g. Chen, 2008; Saxer, 2013), Tibetan medicine in Sichuan (Zhou, 2007), Mongolian medicine in China (e.g. IMAR, 2015; Wu, 2015; Celimuge et al., 2016), Mongolian medicine in Fuxin (Tao et al., 2017; Bai, 2017), and the Tibetan medicine companies Arura and Cheezheng (Wangxiu, 2014). Numerous media articles, mainly from Xinhua and China Daily, as well as company websites (Cheezheng, Arura, Shangri La Tibet Pharmaceuticals, Otaqi, and Hure Pharmaceutical Co.) provided additional, though less reliable, textual sources.

In addition to acquiring data from written sources, we also conducted in-depth semi-structured and open-ended interviews to ascertain the annual sales values of producers whose internal records were inaccessible or did not exist. Since most smaller, private manufacturers produce medicines on a monthly basis and have little overview of their sales value, we generated average monthly production volumes (taking into consideration volatility due to season, weather, and other factors), and the average price per dose of medicine. We also obtained exact numbers of public (provincial and township) and private clinics and hospitals in China through interviews with officials, as well as average numbers of patients-per-day and expenditure-per-patient through direct, on-site observation and conversations with patients and practitioners. This enabled us to calculate the value of the large segment of small-scale, and often not-for-profit, private and public manufacturers, especially in Himalayan India and the rural Tibetan and Mongolian areas of China. Interviews with government officials and producers also yielded comparative data on the relative size of manufacturers, companies and provinces with regard to production volumes and sales values, which – although informal – helped to cross-check other data sources. Even in cases where exact sales values were available, they were always discussed in detail with company officials in order to critically evaluate their credibility. Such interviews revealed that Mongolian producers often underreport sales values; that official Chinese numbers (and those in the media) tend to be inflated; and that the world's largest Sowa Rigpa company, Cheezheng, registers all its income in the Tibetan Autonomous Region even though it is based in Gansu province. Provided such information was supported by other data, as in the above-mentioned examples, we adjusted the numbers accordingly in order to maximize their accuracy. Consequently, not all values presented in this article necessarily match official figures or internal company accounts.

While some of our sources shared information in an official capacity, many others only did so on condition of anonymity and the assurance that their data would only be published in aggregate form. This was especially true with company representatives and government officials in China, and with private Tibetan producers in India. While this article therefore limits itself to the presentation of broad overview data, it is nevertheless based on a wealth of detail, both in terms of company-specific figures and qualitative ethnographic material on local sociocultural, political, and economic contexts. Similarly, all data presented below are derived from at least one, but usually several documented and triangulated sources, even if in many cases we cannot cite them due to research ethics. The entire study was official approved by the Ethics Review Boards of the Austrian Academy of Sciences and the European Research Council, and fully complies with their regulations.

As Martin Saxer (2013: 53) observes, any effort to generate quantitative data on the Sowa Rigpa industry faces a number of serious difficulties, including the lack, inaccessibility, or inconsistency of information. Another challenge is the sheer size and heterogeneity of the industry in China, which defies any attempt at gauging it through classical empirical fieldwork alone. The most fundamental problem, however, is the question of what constitutes the Sowa Rigpa industry in the first place. Asian medical industries – Sowa Rigpa included – do not have stable, commonly agreed-upon boundaries, which means that there are multiple ways of calculating their size and shape. In this study, we are concerned with the *sales value* of both classical prescription formulas *and* non-classical over-the-counter (OTC) health products based on Sowa Rigpa's standard texts and *materia medica*. In geographical terms, our focus is limited to China, India, Mongolia, and Bhutan, the only countries in Asia where Sowa Rigpa has both official recognition and a sufficient degree of industrial development. Furthermore, our analysis does not include data on research and development (R&D) investments, market promotion, charitable activities, or profitability, which constitute promising avenues for further research. The trade in *yartsa gunbu* (*ophiocordyceps sinensis*), a caterpillar fungus highly valued for its health and aphrodisiacal properties, constitutes an industry in and of itself (Winkler, 2009; Dong et al., 2015; Sulek, 2019), and is therefore not considered here, except where it constitutes an ingredient in Bhutanese Sowa Rigpa products. Similarly, we did not consider Sowa Rigpa's indirect generation of value and employment upstream and downstream of its pharmaceutical industry, such as the large raw materials industry, the retail market of Sowa Rigpa products, or the revenues generated through medical college fees. In short, the transnational Sowa Rigpa industry in a broader sense is much larger than the *pharmaceutical* industry this study focuses on. Unless otherwise referenced, all the data presented below are the outcome of original research conducted by the authors. Considering the complexities and limitations of such an endeavor, this study cannot claim to be 100 per cent complete and exact. Despite an inevitable margin of error, however, the bigger picture it reveals of the emerging Sowa Rigpa industry is clear.

## 2 The Sowa Rigpa pharmaceutical industry in context

**Fig. 1:**
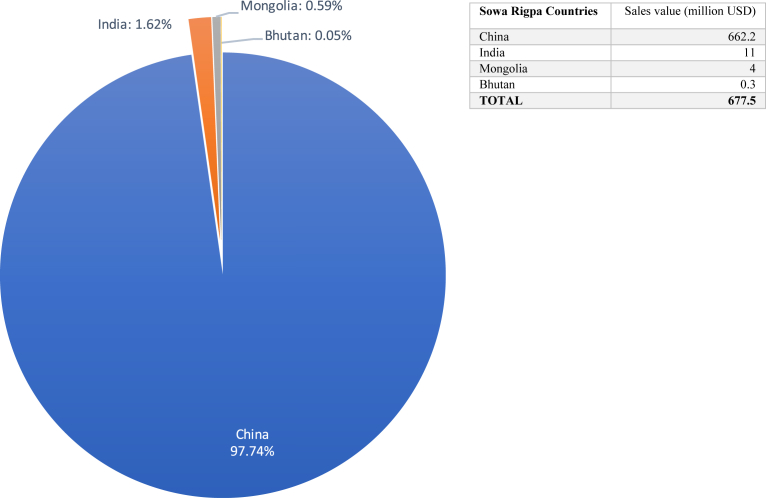
The Sowa Rigpa Industry in Asia 2017, by country.

In 2017, the Sowa Rigpa pharmaceutical industry in Asia, including both prescription drugs and OTC products labeled as Tibetan, Mongolian, Bhutanese, Amchi or Sowa Rigpa medicine, had an annual sales value of 677.5 million USD. Besides an unknown number of retail pharmacies especially in mainland China, more than 2000 clinics and hospitals in the study area – ranging from single rooms in private homes to large hospitals – provided Sowa Rigpa medicines and treatments either exclusively or in combination with other medicines (e.g. biomedicine or TCM). This makes it the sixth largest “traditional” medicine industry in Asia in terms of economic value, after TCM, Ayurveda, Miao medicine, Japanese medicine, and Korean medicine. As shown in Fig. 1, China controls 97.74 per cent of the Sowa Rigpa industry in Asia, generating an annual sales value of 662.2 million USD in 2017. In the same year, India's output was 11 million USD or 1.62 per cent, while Mongolia produced about 4 million USD and Bhutan 308,000 USD, accounting for 0.59 per cent and 0.05 per cent respectively.

There are also Sowa Rigpa traditions in Nepal (Sherpa, Mustang, Dolpo, and the Tibetan exile community in Kathmandu) and parts of Russia (Buryatia, Chita oblast, Tuva, Kalmykia, and more recently St. Petersburg, Moscow, and the Altai region). The data we obtained during visits to Nepal and Russia indicates that Sowa Rigpa is currently undergoing rapid development in all these regions, often coupled with efforts to obtain official state recognition (see also Chudakova, 2015a,b; Blaikie & Craig forthcoming). However, while Sowa Rigpa plays important social, cultural, and public health roles and is becoming increasingly economically attractive, local medicine manufacture remains too individualized, small-scale, and unofficial to be considered an industry in the context of this article.

## 3 The Sowa Rigpa Industry in China

How can China's dominance be explained? History and demographics certainly play important roles, as Sowa Rigpa's historical heartland is located in modern day China and roughly 72 per cent of the world's population with Sowa Rigpa traditions (Tibetans and Mongolians) reside there. China's recent economic transition is also crucial, as liberal market policies have led to unprecedented growth rates and a resurgent private sector, propelling the country to a leading position not only in Asia but globally. Within this larger context, Chinese national and provincial policies regarding “traditional” medicines – and particularly Sowa Rigpa – provide the key to understanding the latter's dramatic growth and industrialization over the past 20 years. After existing Tibetan and Mongolian medicine infrastructures had been seriously damaged by Communist reforms and the Cultural Revolution, both were singled out for state-supported development in the wake of Deng Xiaoping's 1978 political and economic liberalization (Hofer, 2018). As a consequence, the 1980s saw the founding of many new Tibetan (Li, 2012) and Mongolian (Bai, 2017; Tao et al., 2017) medicine hospitals and colleges, and a growing scale of medicine production, which after China's shift towards a “socialist market economy” in 1992 began to produce for profit and a wider market. Against the backdrop of China's 2001 WTO entry, new economic policies in the late 1990s and a new Drug Administration Law in 2001 introduced a nationwide system for drug registration, and mandated the implementation of Good Manufacturing Practices (GMP) for all commercial pharmaceutical producers by 2004 (Saxer, 2013: 35).

In 2007, an eleven-bureau Central Government committee issued a joint initiative to strengthen “ethnic medicines” through interventions in the fields of health, education, infrastructure development, technology, finance, labor, food and drug policy, and intellectual property administration. This initiative was made policy in the 17th National Congress, and further developed in 2012 during the 18th Congress. In 2016, based on these policies, China's first law on TCM was passed, a legal framework within which all ethnic medicines were subsumed and supported. This law instituted equal emphasis on ethnic and Western medicines, with central government support on multiple levels. In combination, since the 1990s these policies led to focused development and the privatization of hitherto government-run Tibetan and Mongolian pharmaceutical enterprises, as well as the foundation of new profit-oriented companies, creating the conditions for the full-scale industrialization of Sowa Rigpa. These government-supported institutions and private companies employed advanced industrial modes of production that significantly increased their output, and by complying with national safety and quality standards and drug registration laws, also gained unprecedented access to China's huge domestic market.

**Fig. 2:**
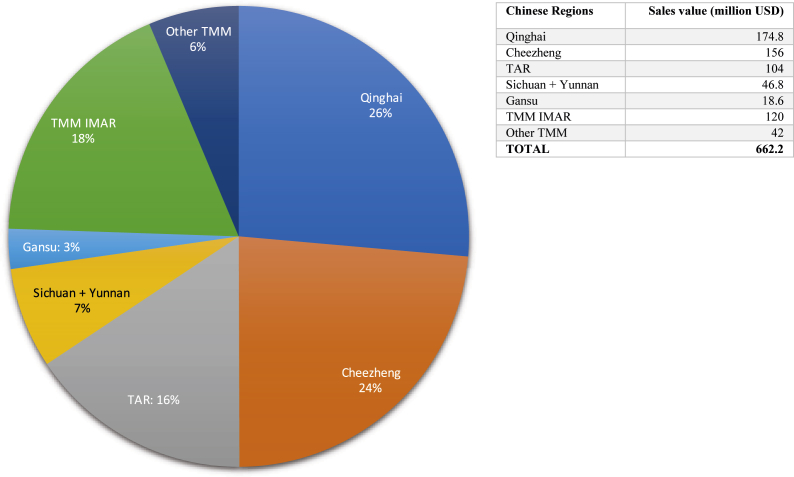
The Sowa Rigpa industry in China 2017, by region.

Since the early 2000s, the Tibetan and Mongolian medicine industries have become China's most important medical resources after biomedicine and TCM, constituting the economic and public health backbone of entire regions. Thus, according to unpublished official numbers, there were 281 Tibetan and 94 Mongolian medicine hospitals operating at various grades and levels (e.g. “international”, national, provincial, county, city) in China, including the three largest ethnic medicine hospitals in the country: the “Inner Mongolia International Mongolian Medicine Hospital” in Hohhot with 1500 beds, the Qinghai Provincial Tibetan Medical Hospital in Xining with 700 beds, and the Men-Tsee-Khang Tibetan Medicine Hospital in Lhasa with 500 beds. In addition, there exist over 1000 public and private Tibetan medicine clinics, and over 300 Mongolian medicine clinics in China. All hospitals and a significant number of the clinics manufacture their own medicines according to “Good Production Practices” exclusively for internal, non-commercial use. In contrast, 54 Tibetan and 25 Mongolian pharmaceutical companies (Celimuge et al., 2016) follow stringent (and expensive) national Good Manufacturing Practices (GMP) and drug registration regulations, which enable them to commercially sell their medicines to other clinics and retail pharmacies in their province or nationwide, depending on their licenses. Overall, the sales value of Tibetan and Mongolian medicines produced in 2017 in China was 500.2 and 162 million USD respectively, amounting to a total of 662.2 million USD. Given the size of China's Sowa Rigpa industry, it is interesting to break it up further by province and medical tradition (Fig. 2).

**Fig. 3:**
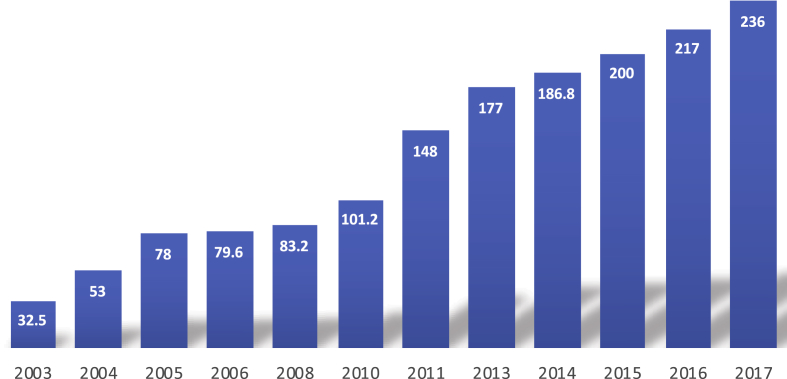
Development of the Sowa Rigpa industry in the TAR (in million USD).

According to high-level official sources, the historical heartland of Tibetan medicine, the Tibetan Autonomous Region (TAR), produced Tibetan medicines worth 217 million USD in 2016, and about 236 million USD in 2017. These numbers cover the TAR's commercial sector of Tibetan medicine consisting of 18 GMP-certified producers, including Cheezheng, Ganlu Pharmaceuticals in Lhasa (the commercial offshoot of the Lhasa Men-Tsee-Khang's factory), Zangnuo Pharmaceuticals in Shigatse (formerly Shigatse Men-Tsee-Khang's factory), Shongpalhachu Shenshui Tibetan Pharmaceuticals near Lhasa, and Tibet Shenhou Pharmaceuticals in Nyalam. Longitudinal data (Fig. 3) show that this sector has more than doubled in size between 2003 and 2005, and then again between 2010 and 2015, illustrating the general trend of development and growth in the TAR's Sowa Rigpa pharmaceutical industry.

While these data illustrate the general growth of the Sowa Rigpa industry in the TAR, they do not consider non-commercial Sowa Rigpa producers like the prestigious Lhasa Men-Tsee-Khang hospital, several dozen county hospitals, a larger number of township clinics, monastic establishments, and small private clinics and manufacturers, which together produced an estimated sales value of 24 million USD in 2017. On the other hand, they include Cheezheng Industry Group, which registered its entire income in the TAR even though its headquarters and main production site are located in Gansu, a different province. In order to avoid distortion and enable more accurate comparison, therefore, we subtract Cheezheng from the TAR figures (counting it as a separate entity, except in Fig. 3), but add the above-mentioned non-commercial sector, leaving a corrected total sales value of 104 million USD for the TAR.

Cheezheng Industry Group is best known for its pain patches and other OTC health products, but also produces a wide range of classical Sowa Rigpa formulas, which it distributes to a large number of clinics and hospitals in Gansu, Qinghai, TAR and Sichuan. It was registered in 2009 on the Shenzhen Stock Exchange and holds over 100 patents on Tibetan medicine, giving it unrivaled market access across China. Its total sales value was 156 million USD in 2017 (147 million USD in 2016, and 176 million USD in 2018), making it by far the largest Sowa Rigpa producer in the world.

Although home to a Tibetan population less than half the size of that in the TAR (SBQP, 2018), Qinghai Province holds a dominant position in the field of Sowa Rigpa today. Arura Group and Jiumei Tibetan Medicine, two of the most prominent corporate Sowa Rigpa producers in the country, are located in Qinghai's capital Xining, and together with 13 other commercial Sowa Rigpa producers like Deumar, Jintro, and Gesar generated a total sales value of 77.4 million USD in 2017. Equipped with GMP certificates and numerous drug licenses, these companies are able to cater not only to rural Tibetan populations, but also to the mainland Chinese market. Twenty private hospitals (total 8.8 million USD) and 230 private clinics (calculated at an average of 15 patients/day spending 200 CNY: total 34 million USD), who may or may not be profit oriented, mostly cater to local populations, as does Qinghai's large public sector of Tibetan medical institutions and producers. This sector is led by the Qinghai Provincial Tibetan Medical Hospital (16.3 million USD), but also includes 26 other government hospitals (average 30 patients/day spending 200 CNY: total 8.3 million USD) and 188 township clinics (average 15 patients/day spending 200 CNY: total 30 million USD). Together, these public institutions generate 54.6 million USD, or 31 per cent, of the province's overall sales value of 174.8 million USD.

Over the past few decades, Qinghai's Tibetan medicine industry outperformed its counterpart in the TAR, so that in 2017 its sales value was significantly higher than the latter, excluding Cheezheng. This was mainly due to comparatively liberal socioeconomic policies implemented in Qinghai Province, which enhanced the local impact of centrally subsidized, large-scale development initiatives during China's reform years. The “Open Up the West” campaign launched in 2000 (Goodman, 2004) was particularly important in this regard, making Qinghai the focus of many regional development policies and domestic private investors. Qinghai's administrative organization (85 per cent of the province are Tibetan-led prefectures and counties), too, allows for greater political and economic flexibility and regional mobility, which facilitates trade, business development, and market access.

Despite comparatively small Tibetan populations, the neighboring province of Gansu is home to important Tibetan medical institutions and producers, and a good number of public and private clinics. As mentioned, Cheezheng's headquarters and main production site are located in Gansu (Lanzhou and Dzö), operating and supplying a large number of clinics in the region. Gansu is also the site of Labrang monastery's historical Tibetan medical school and pharmaceutical factory, some other private companies (e.g. Khagya, Phokö; total 8.7 million USD), Dzö Men-Tsee-Khang, seven county hospitals (total 2.5 million USD), and 47 township clinics (calculated as in Qinghai: total 7.4 million USD). In sum, Tibetan medicine producers in Gansu thus generated a sales value of 18.6 million USD in 2017, excluding Cheezheng.

The Tibetan areas of Sichuan and Yunnan (Kham) are well known as a major source for medicinal herbs as well as the seat of Tibetan medicine's “Southern School” in Derge. In Sichuan, nine commercial pharmaceutical producers (including Dzongsar, Shitsang, Jinzhu, Yuthok, and Norbu Khang) generated a total sales value of about 10 million USD in 2017, with several of the smaller ones (e.g. Dzogchen) just beginning major expansions that year. Thirty-two government (county and city) hospitals including important institutions in Derge, Dartsedo (Ganze), and Dzorgey County (total 19 million USD), some 100 township clinics (calculated as in Qinghai, total 14.5 million USD), and an unspecified number of private clinics (estimated by local sources at 2.5 million USD total) contributed another 36 million USD. In Yunnan, which only has a very small Tibetan population, there are two public Tibetan medicine hospitals (Shangri la and Dechen) and one private commercial producer (Tibet Pharma Co, formerly Shangri la Tibetan Pharmaceuticals), which after bankruptcy and a change of ownership resumed production of 5 formulas on a small scale in 2017. While particularly Tibet Pharma has ambitious plans for growth in the medium-term future, Yunnan's Sowa Rigpa pharmaceutical industry is comparatively insignificant with about 800,000 USD sales value generated in 2017. The total sales value of the Sowa Rigpa pharmaceutical industry in Sichuan and Yunnan thus amounts to 46.8 million USD.

Since the 1950s, “Traditional Mongolian Medicine” (TMM) in China is considered a separate “minority” or “ethnic” medicine from Tibetan medicine (Saijirahu, 2007), although it clearly belongs to the Sowa Rigpa family (Janes and Hilliard 2008). While it has a highly developed hospital infrastructure, its pharmaceutical industry is smaller in both value and profile than that of Tibetan medicine, with significantly lower prices. In the Inner Mongolian Autonomous Region (IMAR), 18 commercial TMM companies, including Inner Mongolia Mongolian Pharmaceuticals in Tongliao (the largest producer), Ulanhot Zhongmeng Pharmaceuticals (part of the Otaqi Group), and the Inner Mongolia Hure Mongolian Medicine (the country's oldest TMM company), generated about 86 million USD. Together with 54 non-commercial production facilities for internal use in hospitals/clinics (total 32 million USD) and an unknown number of small private clinics (estimated at 2 million USD), the 2017 sales value of the TMM pharmaceutical industry in the IMAR was 120 million USD. An additional 42 million USD was generated by seven companies (including Fuxin Mengyao Pharmaceuticals) and a smaller number of hospital factories and private clinics in the Mongolian areas of Liaoning, Jilin, Qinghai, and the Xinjiang Uighur Autonomous Region. In total, TMM thus contributed 162 million USD, or 24 per cent, to China's Sowa Rigpa industry.

## 4 The Sowa Rigpa industry in India

While in China, state policies pushed Sowa Rigpa into the market, it was the other way around in India. Here, it was market forces – that is, Sowa Rigpa's increasing economic value and its resulting legibility to the state – that pushed it, belatedly, into the policy domain. Sowa Rigpa had been practiced at India's Himalayan margins in Ladakh, Zangskar, Lahaul, Spiti, Sikkim, and Tawang for centuries (Pordié & Kloos forthcoming), and was institutionalized and globalized by the Tibetan exile community there since the early 1960s (Kloos, 2008). Yet, it remained largely ignored and unregulated by the Indian government until increasing commercialization in the 1990s and 2000s led to its official recognition in 2010 (Kloos, 2016; Blaikie, 2016). Indeed, while Sowa Rigpa's growth and development in India largely occurred in the absence of state involvement, it was precisely its growing public demand and economic promise that finally secured its legal recognition in the Indian health care system.

**Fig. 4:**
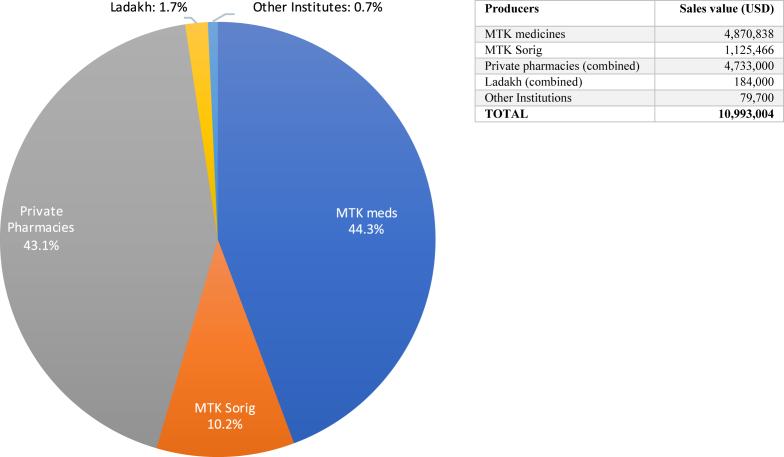
The Sowa Rigpa industry in India 2017.

In 2017, the Indian Sowa Rigpa pharmaceutical industry had a total sales value of about 11 million USD (see Fig. 4), to which the Dharamsala Men-Tsee-Khang (MTK) – the largest Tibetan medical institution outside China – contributed almost exactly 6 million USD, or 54.5 per cent. MTK employed a workforce of 588 (144 of which were doctors) in its pharmacy, medical college, administrative and research departments, branch clinics (53 in India and 3 in Nepal), and various assignments outside of South Asia. The other major force were ten private Tibetan producers of varying size, most of them located in the greater Dharamsala area and in Delhi, who produced medicines worth 4.73 million USD in total or 43.1 per cent (calculations based on detailed data on production volumes and average price per dose in private clinics), either for their own use or to supply private clinics in India and abroad. In addition to MTK and other institutions, there are an estimated 30 private clinics in India, including those of producers.

In Ladakh and Zangskar, the single biggest manufacturer is the Ladakh Amchi Sabha, which in 2017 sold medicines worth about 48,400 USD to official Primary Health Centers (Blaikie, 2019), followed by five individual producers who supplied medicines worth 43,400 USD to other practitioners. Most of the 120–140 *amchi* (Sowa Rigpa practitioners) in Ladakh, who form the backbone of rural health care in the region, produce at least some of their own medicine, at an estimated combined value of 92,200 USD annually. The total value of non-Tibetan Sowa Rigpa medicine in India was therefore roughly 184,000 USD, considering that no commercial medicine production exists among other Himalayan populations in Lahaul, Spiti, Sikkim, or Tawang.

Finally, there are Indian government-administrated institutions like the CIHTS Department of Sowa Rigpa in Sarnath and the CIBS Department of Sowa Rigpa in Choglamsar (Ladakh), and Tibetan-operated medical institutions like the Chagpori Tibetan Medical Institute in Darjeeling, the new Tibetan medical school and production unit at Palpung Sherabling Monastery in Bir, and the new Sorig Bumshi Menri-Ling school and production unit in Solan (both Himachal Pradesh). Although they all play important social, educational, and public health roles, and produce medicines for a small number of clinics (with ambitious plans of expansion), their pharmaceutical output in 2017 remained economically insignificant, at a combined value of 79,700 USD.

From the very beginnings of Sowa Rigpa's industrialization in India in the late 1990s, the demand for medicines (both within and outside MTK) has consistently outstripped the supply. While this large growth potential initially led to the establishment of private producers, its persistence also reveals the industry's most important limiting factor. Despite the undisputed profitability of Sowa Rigpa production in India, few practitioners have the required professional expertise, entrepreneurial spirit, and personal commitment to choose this specialization over practicing in a clinic with fixed working hours. Nevertheless, private producers have been steadily gaining market shares over the past two decades, effectively breaking the MTK's earlier de-facto monopoly on medicine production (Kloos, 2008). Having started from zero, their combined sales value of classical formulas has become a serious market force (4.73 million USD vs. the MTK's 4.87 million USD), underscoring the important role played by the private sector.

**Fig. 5:**
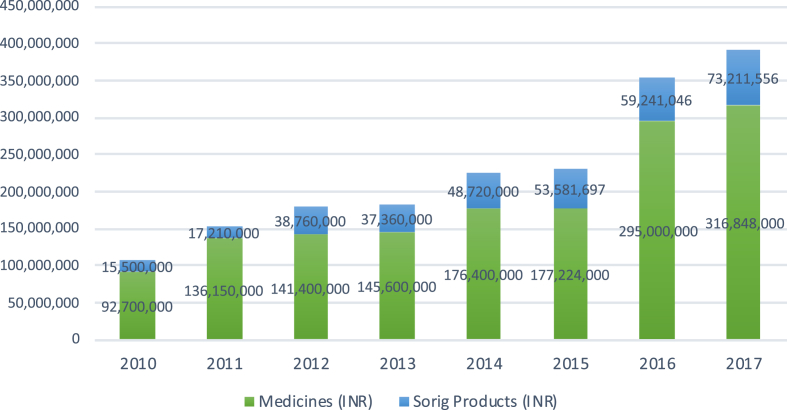
Growth of classical medicines, herbal products, and overall sales value at MTK, 2010-17.

Still, MTK remains not only the largest producer by far, but also the market leader in Sowa Rigpa herbal health products (teas, massage oils, tonics and cosmetics, branded as ‘Sorig products’), which added another 1.125 million USD to its total annual sales value. In order to correctly display longitudinal data of MTK to illustrate growth rates undistorted by annually increasing INR-USD exchange rates, Fig. 5 displays INR values. Thus, the sales value of classical medicines more than tripled (from 92.7 million to 316.8 million INR), due to not only increased production but also significant increases in the average price per dose (3 INR in 2010–11, 3.5 INR in 2012–15, 5 INR in 2016, and 5.52 INR in 2017). The value of Sorig products increased almost five-fold (from 15.5 million to 73.2 million INR). For the period between 2010 and 17, the MTK's overall growth rate of 260 per cent is thus higher than that of the TAR with 133 per cent, where the average CNY-USD exchange rates of 2010 and 2017 are the same.

While India's Sowa Rigpa industry is the second largest after China, it clearly remains at an early stage of industrial development, in large part due to the much more recent advent of official recognition and external support. Dominated by one large institution at times unsure about its role and direction, and held back by a lack of national policies and regulatory frameworks as well as a host of other structural factors, it has little hope of catching up, let alone directly competing, with its counterpart in China. Nevertheless, given ubiquitous plans for expansion, newly available government funds, and Sowa Rigpa's popularity in India's huge domestic market, the Indian Sowa Rigpa industry is likely to grow substantially over the next ten years. While Tibetan institutions and producers, who developed the Sowa Rigpa industry in India in the first place and generated 98.3 per cent of its sales value in 2017, will continue to lead the field, Indian Himalayan Sowa Rigpa practitioners will benefit disproportionally from this growth, as government positions and funding schemes favor Indian citizens (Blaikie, 2016).

### 4.1 The Sowa Rigpa industry in Mongolia

In Mongolia, we observe a mixed scenario, as state policies since the 1990s have aimed to develop Mongolian medicine both as a national public health resource and as a profit-oriented industry (Mongolian Ministry of Health and School of Pharmacy, 2007). Sowa Rigpa is officially integrated into Mongolia's national health system as “Traditional Mongolian Medicine” (TMM), and is covered by national health insurance. A new drug registration system was successfully introduced by 2017, the first national Mongolian medicine pharmacopoeia was published the same year, and GMP will be mandatory for all producers by 2020. However, frequently changing governments seem to lack the stability, resources and political will to back up such initiatives with significant financial investment, thus partially rendering an otherwise impressive policy structure as a burden – rather than a support – for struggling producers. With an official sales value of slightly over two million USD in 2017 for classical formulas, and an estimated total sales value of about 4 million USD including unofficial producers and commercial herbal products, the Mongolian TMM industry remains underdeveloped. While the overall share of the TMM industry (Mongolia and China combined) in Asia's Sowa Rigpa industry is 25 per cent, Mongolia accounts for only 2.4 per cent of that share, and 0.6 per cent of the Sowa Rigpa industry's overall value.

**Fig. 6:**
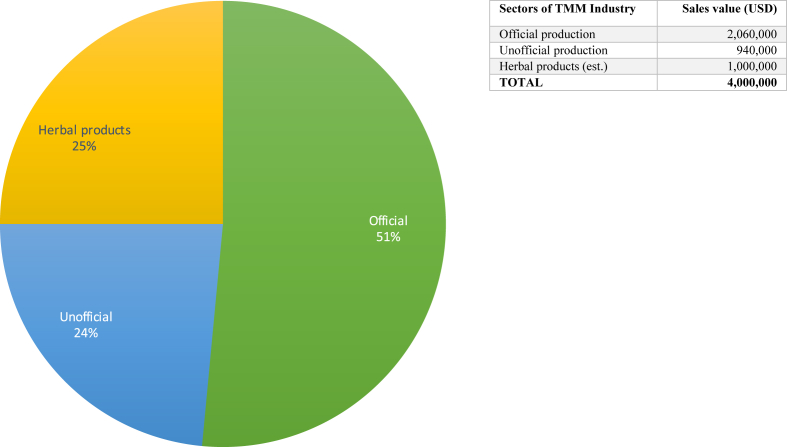
The Sowa Rigpa industry in Mongolia 2017.

The Mongolian Sowa Rigpa industry can be divided into three parts: registered pharmaceutical companies, unofficial manufacturers, and the OTC herbal products sector (see Fig. 6). They are partially overlapping because registered TMM pharmaceutical companies tend to underreport their sales values, making part of their production unofficial, or because they also produce OTC herbal products that do not figure in statistics on classical TMM prescription drugs. There were eight registered TMM producers in 2017, all based in the capital Ulaanbaatar, only one of which – the Institute of Traditional Medicine and Technology (ITMT, formerly “Traditional Medical Science, Technology and Production Corporation”) – was government-owned and operated (see also Pitschmann et al., 2013). The other seven private companies included Mong-Em, Manba Datsan, Oditan, Ariun Mongol (Armon), the Liver Disease Center, and the Traditional Medicine and Herb Co. Together, they produced 2.06 million USD worth of classical medicines (HDA & WHO, 2017), following official drug registration laws and pharmaceutical quality control regulations. In addition to them, one bigger producer (comparable to a medium-sized official company) and about 50–60 small manufacturers produced an estimated 940,000 USD worth of medicines for their own clinical use (an estimate of unreported sales by official companies is included here). While their clinics may be registered, their production facilities are not, enabling them to produce more cheaply by evading costly government regulations, but barring their access to domestic and export markets (reminiscent of non-commercial producers in China). Finally, there is the OTC herbal products sector, led by companies like Oditan (Dr. Baatar brand), ITMT, and Monos (the country's biggest pharmaceutical company with an active interest in TMM products). Having been unable to obtain official data on the size of this sector, based on our knowledge of the three above-mentioned companies we calculated an estimated annual sales value of one million USD for 2017.

Due to incongruent and conflicting official statistics (Mongolian MoH, 2007; 2012; HDA/WHO, 2017), it is not possible to trace the Mongolian TMM industry's longer-term development in any detail. However, combining such statistics with observational and interview data, it is clear that the TMM industry's growth in value was closely connected to two major factors. The first, as in China, was state policy support, such as the mandatory integration of TMM facilities in all second and third level public hospitals, which fueled a demand for both TMM graduates and medicines. According to older official numbers (Mongolian MoH, 2007; 2012) and newer interview data from health officials, there were 175 hospitals and clinics offering TMM in 2014, as compared to 140 in 2012, and 115 in 2007. In practice, however, “traditional medical services” offered especially in public biomedical hospitals may include anything from acupuncture, electricity treatment, light, water, oil, and mud therapies, massage, bloodletting, moxibustion, cupping, medicinal herbs, to physical exercise (Mongolian MoH, 2012: 23). TMM's actual coverage, according to practitioners and experts, is only 2–3 per cent of all health care in Mongolia, which is also TMM's market share in Mongolia's pharmaceutical industry (HDA & WHO, 2017). At the same time, Mongolia's TMM education infrastructure is comparatively large and profitable, with six TMM colleges or university departments, over 2000 students, and some 500 graduates in 2017 alone.

The second major factor in the development of the TMM industry was the Mongolian economy overall, the boom-and-bust cycles of which corresponded with phases of growth and decline of the TMM industry. Thus, the latter's size roughly tripled between 2001 and 08, contracted in 2009 during a general economic crisis, almost quadrupled again during the boom years of 2010–14, only to be hit hard by another economic crisis in 2015–16. To a large extent, this vulnerability to general economic fluctuations stems from a lower insurance coverage for TMM (compared to biomedicine) under the national health care system, which makes TMM a less attractive option for cash-strapped patients, especially in difficult times. Although Mongolia's political situation barely improved, in 2017 TMM entered another period of substantial growth, as two new companies were founded and most existing producers began to expand their operations, either through an increasing focus on OTC herbal products (e.g. Oditan) or by strengthening their export markets (e.g. Manba Datsan, Armon, ITMT). The most important export destination for TMM medicines was Poland, which hosts some 200 active TMM practitioners from Mongolia, followed by Russia, Australia, the USA, and other Eastern European countries. Depending on the company, up to 30–40 per cent of the total production was exported. While remaining relatively small, the Mongolian TMM industry is likely to continue its uneven, but overall consistent, path of growth in the medium-term future.

## 5 The Sowa Rigpa industry in Bhutan

In Bhutan, state policies kept Sowa Rigpa outside the market for a long time. Sowa Rigpa officially became part of the national health care system in 1967/68, and was subsequently institutionalized and monopolized in the form of the government-operated and funded National Institute of Traditional Medicine (NITM) (Wangchuk, 2008). Although NITM's pharmaceutical production unit, Menjong Sorig Pharmaceuticals (MSP), began to sell commercial products (incense, herbal teas etc.) in the 2000s and gained more independence as a corporation in 2017, Sowa Rigpa in Bhutan remains first and foremost a national public health resource rather than a profit-oriented business. In 2017, the government operated 57 “traditional” medicine units across the country, employing 75 *drungtshos* (physicians) and 116 *menpas* (medical assistants) prescribing a range of 98 MSP-produced medicines (Sonam, 2017). Bhutan's low annual sales value in Fig. 1 is thus partially the result of its intentionally low medicine pricing policy, as well as its small population of less than 800,000.

**Fig. 7:**
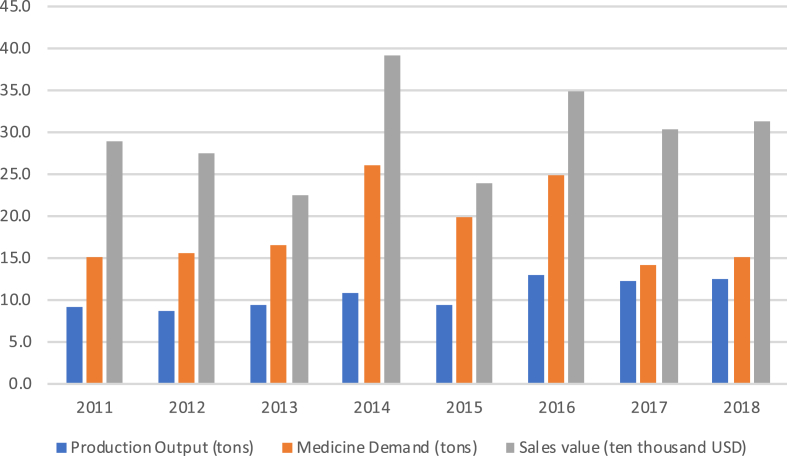
The development of the Bhutanese Sowa Rigpa industry (production, demand, and revenue).

According to MSP's accounts, it had a total sales value of 308,342 USD in 2017, of which about three quarters came from medicines and one quarter from commercial products such as incense, herbal tonics, *yartsa gunbu*, and beauty creams. The Bhutanese numbers fluctuate more than any other country in this study, making it impossible to identify any trend of sustained growth in terms of either production value or volume between 2011 and 2017. MSP's sales value ranged between 224,000 and 347,000 USD, and its production volume – relevant because national demand for Sowa Rigpa medicines is measured in metric tons – between 9 and 13 tons of medicines, which is comparable to an average Tibetan-run private pharmacy in Dharamsala. As shown in Fig. 7, despite Sowa Rigpa being fully integrated into national health care and insurance systems (Choden and Dorji, 2014), until 2016 this output never covered more than 60 per cent of national demand, and in 2014–15 even less than 50 per cent.

**Fig. 8:**
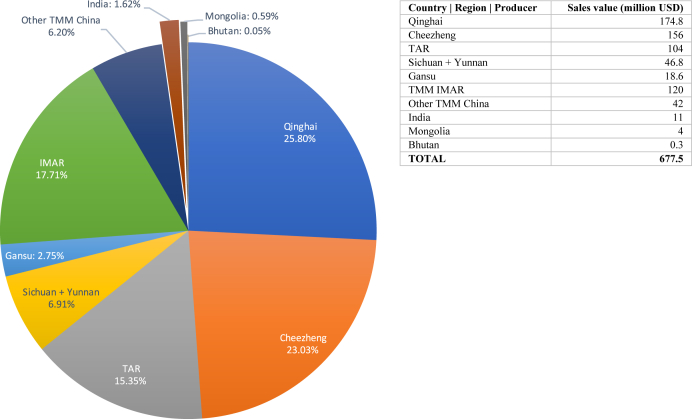
The Sowa Rigpa Industry in Asia 2017, overview with countries and regions.

More recent figures suggest that the hoped-for increase of MSP's productivity after its corporatization has so far remained elusive. Instead, the official numbers on demand have been adjusted to about half of their actual size. On the other hand, this dramatic shortfall also indicates a growth potential of 100 per cent or more, provided MSP manages to increase its production. The growing popularity of Sowa Rigpa among Bhutan's population, a good policy and infrastructure environment for “traditional” medicine, Bhutan's natural abundance of medicinal plants, as well as plans to develop the country as a health tourism destination and efforts to increase exports of OTC herbal products provide favorable conditions for more stable growth rates in the medium-term future.

## 6 Trends and observations

This article provides hitherto non-existent basic information about the size, shape and dynamics of the Sowa Rigpa industry in Asia by compiling original quantitative data from China, India, Mongolia, and Bhutan. Beyond its immediate interest to professionals, industry actors, and policy makers, as a big-picture overview of this industry it lays solid foundations for further academic research into contemporary Sowa Rigpa. A number of broader trends emerge from these data and other sources, which may provide further starting points for comparative investigation. By way of summary, therefore, we outline some major observations and trends that characterize the contemporary Sowa Rigpa industry in Asia and point towards its likely development trajectory in the foreseeable future.

With a size of 677.5 million USD in 2017 (see Fig. 8), the Sowa Rigpa industry constitutes one of the most important economic, health, and cultural resources for Tibetan, Mongolian, and Himalayan communities in Asia. Since the year 2000, the overall size of the industry has multiplied by a factor of ten, and is much larger still if its retail and raw material markets, education sector, hospital care, research and development, or charitable activities are accounted for. While these numbers appear insignificant in the context of Asia's economies overall, they are nothing short of impressive when placed in the context of the total population of Asian regions with Sowa Rigpa traditions. Thus, if the native population base of the TCM and Ayurveda industries counts over one billion each, the transnational Sowa Rigpa industry is rooted in a total population of less than 19 million Tibetans, Mongolians, and Tibetan Buddhist Himalayans – smaller than Beijing, Delhi, or Shanghai, and certainly less affluent or politically powerful. In this context, the Sowa Rigpa industry's role not just as a health care provider, but also as a generator of jobs, economic opportunity, cultural identity, and political power among Tibetan, Mongolian, and Himalayan communities in some of the most marginal areas of Asia is revealed. Regardless of important questions of ownership, which would merit a separate article, the industry strongly depends on uniquely Tibetan expertise and legitimation that ensures the involvement of local communities, and retains a large not-for-profit sector especially in rural areas.

Secondly, our data show that China has by far the largest industry, contributing 97.74 per cent of the total sales value of Sowa Rigpa medicines. However, this has not yet translated into Chinese dominance of Sowa Rigpa's global control, representation, or identity. In this regard, the Sowa Rigpa medical communities in India and Mongolia still have an edge, with large numbers of practitioners working abroad in and beyond Asia, numerous English-language publications on the topic (Rechung, 1973; Donden, 1986, 2000; Dakpa, 2007; Gyatso and Hakim, 2010; Men-Tsee-Khang, 2008, 2011), and a strong presence at international academic events (Kloos, 2017a). Indeed, it was the exile Tibetan medical community that globalized Sowa Rigpa since the late 1970s, and considering their small population size of only 120,000 (as compared to 6 million Tibetans in China), their performance is remarkable: in terms of value generated per capita, they can easily compete with their peers in Tibet. However, Tibetans and Mongolians in China are catching up fast, mainly in the domains of scientific research, the securing of patents and other intellectual property rights, official international recognition, as well as by organizing high-quality academic and professional international conferences.

Thirdly, our data indicate an increasing diversification taking place within the industry, as local, small-scale, not-for-profit Sowa Rigpa institutions coexist with commercially oriented companies and individuals, each carving out their own market niche. Some claim long medical lineages or affiliation with important religious or cultural leaders, others emphasize their monastic status, still others superior quality ingredients, cutting-edge quality control technologies, or simply their size (where either being “big” or “small” can be presented as an advantage – see Blaikie & Craig forthcoming). At this point, it appears that the market is large enough and growing fast enough to accommodate all these diverse actors. However, this diversification is leading to a blurring of boundaries with other medical traditions, including biomedicine. For example, some companies produce Sowa Rigpa, Chinese and Western medicines; others market slightly reformulated Chinese drugs as “Tibetan medicine” and vice versa; and many Tibetan and Mongolian doctors have absorbed therapies like acupuncture or panchakarma into their own traditions. Such fluid boundaries are emerging as key characteristics of Asian medical industries in general (Kloos & Blaikie forthcoming), but make it more difficult to quantitatively gauge them.

Fourth, notable shifts are underway in the kinds of products being made and marketed under the banner of Tibetan/Mongolian/Himalayan medicine. While classical Sowa Rigpa formulas remain popular with patients and are still produced in the largest quantities by the majority of firms, we also observe increasing pharmaceutical innovation in terms of the reformulation of existing medicines, and the development of new formulas and product forms such as tablets, syrups and capsules. Connected to this is a trend towards the development of innovative herbal products derived from Tibetan/Mongolian/Himalayan medical knowledge or based on Sowa Rigpa *materia medica*. Developed in India by MTK, the Sorig Products line of herbal teas, cosmetics and tonics is a good example of this, as are Cheezheng's pain patches, MSP's herbal product lines in Bhutan or, more recently, the Dr. Baatar brand in Mongolia. Such products are much more profitable and easier to produce than medicines, and by evading the strict regulations that apply to drugs, can reach larger national and global markets. In this regard, the Sowa Rigpa industry is following a similar trend to the Indian Ayurveda industry (Pordié, 2014; Pordié and Gaudillière, 2014; Bode, 2015).

## 7 Conclusion

The data presented in this article indicate that national policies are a crucial factor for the growth and dynamics of the Sowa Rigpa industry. While massive state intervention - both political and financial - catalyzed and continues to spur Sowa Rigpa's rapid growth in China (especially in Qinghai), it was the absence thereof that slowed industrial development in India. Government policies also had definite impacts on Sowa Rigpa's development in Bhutan and Mongolia, where integration into public healthcare has fostered sizeable but low-profit domestic markets. Bhutan has been consistently unable to produce sufficient quantities of medicine to meet public demand, despite (or because of) its state-backed monopoly, whereas a wider array of companies in Mongolia struggle to maintain growth and profitability in a context of political and economic instability. State policies can help or harm and often do both at the same time, but they always play crucial roles in shaping Sowa Rigpa's industrial development.

A second crucial factor for the Sowa Rigpa industry's development that emerges from the above data, albeit more indirectly, is the existence of sociocultural structures such as professional, spiritual, and family lineages. Without the specifically Tibetan legitimation gained through affiliation with such lineages and the moral economy of Tibetan Buddhism (Saxer, 2013), and without the expert pharmaceutical knowledge that is connected to it, it remains difficult to participate in this industry, let alone develop it. This is the reason why the industry retains a strong not-for-profit sector, why even Han Chinese billionaire entrepreneurs in the field of Sowa Rigpa find it necessary to seek Tibetan Buddhist legitimation through charitable work and the construction of lineage connections, and why the industry is strongest in the Tibetan Buddhist heartlands. Despite various legal ownership structures, then, Tibetan/Mongolian/Himalayan *social, cultural and intellectual ownership* remains of central relevance to the Sowa Rigpa industry today.

Whether it appears desirable or not, and whether individual practitioners or institutions actively participate in it or not, the Sowa Rigpa pharmaceutical industry has become big enough to transform Sowa Rigpa as a whole. This pertains also to those actors who do not see themselves as directly participating in the industry, or even actively seek to remain outside it. In large parts of Asia, but increasingly also on a global scale, the Sowa Rigpa industry has become impossible to ignore. As a transformative force, it is complex, ambivalent, and multi-faceted, and its social, political, economic, and health consequences will remain a productive field of study for a long time to come. We argue that it can, and should, be better understood, regulated, and supported through careful research, appropriate and progressive policies, and strategies that better balance development with long-term sustainability. While this article constitutes an important first step, further research on various aspects of the Sowa Rigpa industry has a crucial part to play in moving towards these goals.
